# Supplementary material for: Task specialization across research careers
Source: eLife. 2020 Oct 28;9:e60586. doi: 10.7554/eLife.60586 (PMC7647403; doi:10.7554/eLife.60586)
Supplement: Transparent reporting form [file elife-60586-transrepform.pdf]

Westbrook Centre, Milton Road
Cambridge CB4 1YG
UK

P 01223 855340
W elifesciences.org
T @elife

eLife's transparent reporting form

We encourage authors to provide detailed information *within their submission* to facilitate the interpretation and replication of experiments. Authors can upload supporting documentation to indicate the use of appropriate reporting guidelines for health-related research (see EQUATOR Network), life science research (see the BioSharing Information Resource), or the ARRIVE guidelines for reporting work involving animal research. Where applicable, authors should refer to any relevant reporting standards documents in this form.

If you have any questions, please consult our Journal Policies and/or contact us: editorial@elifesciences.org.

Sample-size estimation

- You should state whether an appropriate sample size was computed when the study was being designed
- You should state the statistical method of sample size computation and any required assumptions
- If no explicit power analysis was used, you should describe how you decided what sample (replicate) size (number) to use

Please outline where this information can be found within the submission (e.g., sections or figure legends), or explain why this information doesn't apply to your submission:

The paper uses a dataset of publications from PLoS journals. We identified authors through the author name disambiguation algorithm (referred to in the manuscript by Caron and van Eck, 2014). We then compared the productivity ratios of the authors with all of the authors identified via the same algorithm in Web of Science from 1980 to 2018 (last update of the database at the time). We noted that our sample of authors **is not a representative sample** of all authors in Web of Science, nor in Life and Biomedical Sciences. For this reason we opted to apply our model only to authors included in the first dataset. Furthermore, throughout the paper we refrain to state that our findings are universal and we keep our further analyses at a descriptive level (comparison with length trajectory, gender, productivity and impact, and author order).

Replicates

- You should report how often each experiment was performed
- You should include a definition of biological versus technical replication
- The data obtained should be provided and sufficient information should be provided to indicate the number of independent biological and/or technical replicates
- If you encountered any outliers, you should describe how these were handled
- Criteria for exclusion/inclusion of data should be clearly stated
- High-throughput sequence data should be uploaded before submission, with a private link for reviewers provided (these are available from both GEO and ArrayExpress)

Please outline where this information can be found within the submission (e.g., sections or figure legends), or explain why this information doesn't apply to your submission:

The analyses have been replicated at least 3 times by the corresponding author and code has been revised by last author.

Statistical reporting

- Statistical analysis methods should be described and justified
- Raw data should be presented in figures whenever informative to do so (typically when N per group is less than 10)
- For each experiment, you should identify the statistical tests used, exact values of N, definitions of center, methods of multiple test correction, and dispersion and precision measures (e.g., mean, median, SD, SEM, confidence intervals; and, for the major substantive results, a measure of effect size (e.g., Pearson's r, Cohen's d)
- Report exact p-values wherever possible alongside the summary statistics and 95% confidence intervals. These should be reported for all key questions and not only when the p-value is less than 0.05.

Please outline where this information can be found within the submission (e.g., sections or figure legends), or explain why this information doesn't apply to your submission:

We do not report p-values nor statistical significance tests in our results, we did however, crossvalidate the results of our modelling. We report the classification error ratios in Table 3, page 18. These are discussed and explained in line 126 and lines 454-466.

Regarding the archetypal analysis a thorough description is provided in lines 467 to 511, including decisions which could affect the findings, specifically with the choice on the aggregation method from publications to authors (lines 468 to 484).

(For large datasets, or papers with a very large number of statistical tests, you may upload a single table file with tests, Ns, etc., with reference to sections in the manuscript.)

Group allocation

- Indicate how samples were allocated into experimental groups (in the case of clinical studies, please specify allocation to treatment method); if randomization was used, please also state if restricted randomization was applied
- Indicate if masking was used during group allocation, data collection and/or data analysis

Please outline where this information can be found within the submission (e.g., sections or figure legends), or explain why this information doesn't apply to your submission:

Full account on the description of the datasets are provided in lines 338 to 413. Furthermore, the complete datasets are provided at http://doi.org/10.5281/zenodo.3891055. Permission has been granted as some of the data shared are under proprietary copyright terms.

Additional data files ("source data")

- We encourage you to upload relevant additional data files, such as numerical data that are represented as a graph in a figure, or as a summary table
- Where provided, these should be in the most useful format, and they can be uploaded as "Source data" files linked to a main figure or table
- Include model definition files including the full list of parameters used
- Include code used for data analysis (e.g., R, MatLab)
- Avoid stating that data files are "available upon request"

Please indicate the figures or tables for which source data files have been provided:

Source datasets are available at http://doi.org/10.5281/zenodo.3891055.
